# Evaluation of Live Bacterial Prophylactics to Decrease IncF Plasmid Transfer and Association With Intestinal Small RNAs

**DOI:** 10.3389/fmicb.2020.625286

**Published:** 2021-01-14

**Authors:** Graham A. J. Redweik, Mary Kate Horak, Ryley Hoven, Logan Ott, Melha Mellata

**Affiliations:** ^1^Department of Food Science and Human Nutrition, College of Agriculture and Life Sciences, Iowa State University, Ames, IA, United States; ^2^Interdepartmental Microbiology Graduate Program, Iowa State University, Ames, IA, United States

**Keywords:** *Escherichia coli*, ceca, conjugation, antimicrobial resistance, APEC, siderophores production, chicken

## Abstract

Chicken intestinal *Escherichia coli* are a reservoir for virulence and antimicrobial resistance (AMR) genes that are often carried on incompatibility group F (IncF) plasmids. The rapid transfer of these plasmids between bacteria in the gut contributes to the emergence of new multidrug-resistant and virulent bacteria that threaten animal agriculture and human health. Thus, the aim of the present study was to determine whether live bacterial prophylactics could affect the distribution of large virulence plasmids and AMR in the intestinal tract and the potential role of smRNA in this process. In this study, we tested ∼100 randomly selected *E. coli* from pullet feces (*n* = 3 per group) given no treatment (CON), probiotics (PRO), a live *Salmonella* vaccine (VAX), or both (P + V). *E. coli* isolates were evaluated via plasmid profiles and several phenotypic (siderophore production and AMR), and genotypic (PCR for virulence genes and plasmid typing) screens. P + V isolates exhibited markedly attenuated siderophore production, lack of AMR and virulence genes, which are all related to the loss of IncF and ColV plasmids (*P* < 0.0001). To identify a causal mechanism, we evaluated smRNA levels in the ceca mucus and found a positive association between smRNA concentrations and plasmid content, with both being significantly reduced in P + V birds compared to other groups (*P* < 0.01). To test this positive association between IncF plasmid transfer and host smRNA concentration, we evenly pooled smRNA per group and treated *E. coli* mating pairs with serial concentrations of smRNA *in vitro*. Higher smRNA concentrations resulted in greater rates of IncF plasmid transfer between *E. coli* donors (APEC O2 or VAX isolate IA-EC-001) and recipient (HS-4) (all groups; *P* < 0.05). Finally, RNAHybrid predictive analyses detected several chicken miRNAs that hybridize with pilus assembly and plasmid transfer genes on the IncF plasmid pAPEC-O2-R. Overall, we demonstrated P + V treatment reduced smRNA levels in the chicken ceca, which was associated with a reduction in potentially virulent *E. coli*. Furthermore, we propose a novel mechanism in which intestinal smRNAs signal plasmid exchange between *E. coli*. Investigations to understand the changes in bacterial gene expression as well as smRNAs responsible for this phenomenon are currently underway.

## Introduction

Plasmids are mobile genetic elements that can bolster a host bacterium’s fitness in harsh environments like the gastrointestinal tract by carrying genes encoding AMR (Rolain, 2013), iron acquisition factors (Deriu et al., 2013), and antimicrobial-like products colicin (Gordon et al., 1998). In poultry, many of these factors are commonly found in intestinal *E. coli* and other *Enterobacteriaceae* on narrow-range plasmids like incompatibility group F (IncF) (Johnson et al., 2005, 2007, 2010; Yang et al., 2015). However, these factors may have negative consequences for the animal host, as a transfer of these genes can increase bacterial virulence potential and/or generate AMR pathogens (Johnson et al., 2010). Thus, efforts for mitigating the transfer of these virulence and/or AMR plasmids should be a top priority for both animal agriculture and human medicine. Conjugation inhibitors like unsaturated fatty acids (Getino et al., 2015) have been postulated as a means to reduce plasmid transfer. However, no treatment nor prophylactics (i.e., treatments used to proactively prevent disease) have yet been tested for their effect on plasmid transfer in the gut. Thus, a greater understanding of which host factors influence bacterial conjugation as well as treatments to achieve this are imperative.

One such host factor may be small RNA (smRNA), a class of RNA molecules of less than 200 nucleotides in length. These smRNAs include species like microRNA (miRNA), which have intracellular regulatory and immune functions in plants and animals (Liu et al., 2017; Zhou et al., 2018). In chickens, miRNA expression profiles are augmented during bacterial infections with avian pathogenic *Escherichia* coli (APEC) (Jia et al., 2017), a subset of extraintestinal pathogenic *E. coli* (ExPEC; Mellata, 2013), and *Salmonella enterica* (Chen et al., 2017; Wu et al., 2017), suggesting that miRNA may have implications on resistance to bacterial infections. Recently, Liu and colleagues found that the composition of the gut microbiota was highly dependent on miRNA secreted by host intestinal cells into the lumen (Liu et al., 2016). However, how smRNA may influence bacterial activities like plasmid conjugation is unknown.

In addition to bacterial pathogens, non-pathogenic bacteria like probiotics (Chen et al., 2017) can affect chicken intestinal smRNA levels. Previously, we found that immunization with a live *Salmonella* vaccine reduced *Enterobacteriaceae* in the ceca and feces, regardless of probiotic supplementation (Redweik et al., 2019). The aim of this study was to determine whether live prophylactics affect the level of *E. coli* carrying plasmids and genes encoding AMR and ExPEC virulence factors in the chicken intestine as well as whether this effect is correlated with smRNAs.

## Materials and Methods

### Ethics Statement

Chicken experiments were approved by Iowa State University Institutional Animal Care and Use Committee, log #1-16-8159-G. Animal distress was minimized during experimental procedures by providing enrichments and an open-floor setting (room temperatures ranging 73–75°F maintained via heat lamps) to promote social interactions. Furthermore, room temperatures ranging from 73–75°F were maintained via heat lamps within each pen. Euthanasia techniques (CO_2_ asphyxiation followed by thoracotomy) followed the American Veterinary Medical Association Guidelines (2013).

### Experimental Design and Sample Sizes

Samples in this study were taken from chickens used in previous studies (Redweik et al., 2019, 2020). Briefly, 1-day-old specific pathogen-free White Leghorns (straight run mix of males and females; VALO Biomedia, Adel, IA) fed Purina^®^ Organic Starter-Grower were either orally vaccinated with an RASV alone (VAX), supplemented with a commercial probiotic supplement (*Bacillus subtilis*, *Lactobacillus acidophilus*, *Pediococcus acidilactici*, *Pediococcus pentosaceus*, *Saccharomyces pastorianus*) alone (PRO), or treated with both (P + V). No treatment controls (CON) were included for comparison. Two weeks post-boost, feces were collected from three randomly selected birds per group, homogenized and serially diluted in PBS, and plated on MacConkey agar. Then, 100 random isolates were evenly selected among the three birds per group, streaked onto MacConkey agar, and stored in peptone-glycerol solution at -80°C for further analyses. Ceca mucus, the physiological site for plasmid transfer in the chicken intestine (Poulsen et al., 1994) was collected from birds (*n* = 4–5 per group) at 3 weeks post-boost, flash-frozen, and stored at -80°C.

### Phenotypic Andgenotypic Assays

All isolates were streaked on LB agar (0.1% glucose) and preliminarily screened for *E. coli* conserved gene *uidA* (beta-glucuronidase; Supplementary Table 1) to determine *Enterobacteriaceae* identity (Stromberg et al., 2017). All subsequent analyses solely used *E. coli* isolates to identify plasmid-associated phenotypes and genes associated with APEC.

#### Siderophore Production and Antibiotic Resistance

*E. coli* were picked onto CAS agar plates and incubated overnight (ON) at 37°C. Thereafter, oxidation rings were measured in mm. Additionally, *E. coli* were picked onto LB plates spiked with tetracycline (TC; 15 μg/ml) or streptomycin (50 μg/ml) and incubated ON at 37°C. *E. coli* were determined to be resistant to respective antibiotics via positive growth. All *in vitro* assays were repeated twice for confirmation.

#### Virulence Gene and Plasmid-Typing PCR

Colony PCR was used with each *E. coli* isolate to screen for *iss* (increased serum survival), *iutA* (aerobactin receptor), *iroN* (salmochelin receptor), and *hylF* (hemolysin) virulence genes using the primers mentioned in Table 1 and PCR conditions from respective references (Bej et al., 1991; Johnson and Stell, 2000; Johnson et al., 2007, 2008). Furthermore, to characterize the types of plasmids possessed by each isolate, a previously established multiplex array for 18 incompatibility (Inc) groups found in *E. coli* (Johnson et al., 2007) was performed using conditions as previously described (Supplementary Table 2). Additionally, given that APEC virulence genes are commonly found on plasmids harboring the *cvaC* gene (i.e., colicin V or ColV plasmids), we screened all *E. coli* isolates for the presence of this gene (Supplementary Table 2). All PCRs were repeated twice per isolate for confirmation.

**TABLE 1:** *Escherichia coli* strains and large plasmids evaluated in this study.

*Escherichia coli* strains	Role	Plasmid	Plasmid size (kb)	Inc group, relevant antibiotic resistance	References
APEC O2	Donor	pAPEC-O2-R	101	IncF, Tetracycline	Johnson et al., 2006
		pAPEC-O2-ColV	180	IncFIB, N/A	Johnson et al., 2005
IA-EC-001	Donor	IA-P-001A	125	IncF1B, Tetracycline	This study
		IA-P-001B	102	IncI1, N/A	
HS-4 Nal^*r*^	Recipient	None	N/A	Nalidixic acid (spontaneous)	Ott et al., 2020
39R681	Ladder	–	147	N/A	Mainil et al., 1992
		–	65	N/A	
		–	35.85	N/A	

*The symbol “–” indicates that plasmids are not officially named.*

#### Large Plasmid Profiling

Large plasmid content was determined in each isolate as previously described (Kado and Liu, 1981) with minor modifications. Briefly, each fecal isolate was cultured in 4 ml LB broth (0.1% glucose) ON at 37°C, and 1 ml per culture was centrifuged at 10,000 × g at RT. For isolates from conjugation experiments (see section “RNAHybrid miRNA Target Predictions”), transconjugants (i.e., TC_*R*_NA_*R*_) were cultured in 4 ml LB broth (0.1% glucose, 15 μg/ml TC, and 30 μg/ml NA) ON at 37°C. Pellets were resuspended in 200 μl 1X TAE (pH 7.9) and 400 μl lysis buffer (0.02 M Tris, 0.4% SDS, 0.2 M NaOH; pH 12.7). Suspensions were gently mixed and incubated at 37°C for 50 min. Following incubation, 600 μl 1:1 phenol chloroform was added per isolate suspension, gently mixed, then centrifuged for 15 min at 10,000 × g. The subsequent supernatants that contain plasmids were then loaded into a 0.5% TAE gel and ran via gel electrophoresis (40 V) for 860 min at 4°C. Gels were then stained via ethidium bromide and imaged via Azure Imager c300. *E. coli* reference strain 39R681 (Mainil et al., 1992) was used as a ladder to measure plasmid sizes as done previously (Mellata et al., 2010), which were calculated via GelAnalyzer 19.1 software.

### Ceca smRNA Extraction and Quantification

Total smRNAs were extracted from ceca mucus (*n* = 4–5 per group) following mirVana^TM^ miRNA isolation kit (Ambion^®^) following the manufacturer’s procedure for smRNA purification. Subsequently, smRNAs were further purified using Amicon^®^ Ultra-0.5 Centrifugal Filter Devices (Millipore) as described previously (Liu et al., 2016). As calculated by NanoDrop 2000, the A260/A280 ratios for all extracts were approximately 2.0, indicating an acceptable purity of the smRNAs. smRNA quantity was calculated via Qubit^TM^ microRNA Assay Kit (which broadly detects small RNA molecules), and concentrations were calculated by adjusting for the weight (g) of each sample.

### Inter-*E. coli* Conjugation-smRNA Assays

The APEC strain APEC O2 (TC_*R*_ carried on IncF plasmid pAPEC-O2-R (Johnson et al., 2005) was used to model the effect of ceca smRNA on transfer of IncF plasmids to the recipient commensal *E. coli* HS-4 (NA_*R*_) (Table 1). Respective *E. coli* strains were cultured in LB broth (0.1% glucose) with appropriate selective antibiotics ON at 37°C, shaking at 225 rpm. The following day, cultures were adjusted to OD_600_ ∼0.1, centrifuged, and washed with LB broth (0.1% glucose) twice to remove antibiotics from original cultures. Afterward, APEC O2 and HS-4 were mixed 1:1, and 20 μl were added to each reaction on a 96-well plate.

Total smRNA extracted from chickens was pooled by group, serially diluted in LB broth (0–50 ng per reaction), and 180 μl of solutions were aliquoted to respective reactions in duplicate. Conjugation assays were then incubated for 6 h at 41°C, and reactions were serially diluted in PBS and plated on MacConkey agar with various antibiotic compositions (15 μg/ml TC only; 30 μg/ml NA only; 30 μg/ml NA + 15 μg/ml TC) to enumerate donor, recipient, and transconjugant *E. coli*, respectively.

To determine if smRNA-effects on IncF plasmid transfer were not specific to pAPEC-O2-R plasmid transfer, a VAX *E. coli* isolate (IA-EC-001; TC_*R*_; Table 1) was tested as a donor strain in separate conjugation assays. All conditions were kept the same except for an extended incubation for 10 h at 41°C, as IA-EC-001 was determined to optimally transfer TC_*R*_ at this time.

### RNAHybrid miRNA Target Predictions

The predictive binding of chicken miRNAs to the IncF plasmid pAPEC-O2-R genes was accomplished using the RNAHybrid pipeline as described previously (Rehmsmeier et al., 2004; Krüger and Rehmsmeier, 2006). Briefly, the complete DNA sequence of the APEC O2 IncF plasmid pAPEC-O2-R (NC_006671.1) was obtained from NCBI. The *Gallus gallus* specific miRNA library was then obtained by downloading the mature miRNA database (v22) from miRbase^1^ and extracting all sequences with the header text “gga-miR-.” Predictive miRNA targets were determined by binding structure stability and sequence alignment outputs using RNAhybrid^2^ with the default settings. Alignments generating *P*-values less than 0.001 were considered significant bindings for further description. RNAhybrid alignments resulted in reported minimum free energy (MFE, kcal/mol) values with increasingly negative numbers indicating greater affinity binding. Full predictions are summarized in Supplementary Table 3.

### miRNA RT-qPCR and Mimic *in vitro* Assays

To confirm the presence of miRNA species from the top five host miRNA—IncF target gene interactions (see Table 2), total smRNA from each group was converted to cDNA via reverse transcription using the TaqMan Advanced miRNA cDNA Synthesis Kit. Thereafter, custom qPCR primers for each miRNA species were developed via TaqMan Advanced miRNA Assays, and qPCR was performed on StepOnePlus as per manufacturer instructions.

**TABLE 2:** Top five predictive miRNA hybridizations and gene targets of chicken miRNA on the plasmid pAPEC-O2-R.

miRNA (gga-miR-)	MFE (kcal/mol)	miRNA function; reference	Target gene	Gene function; uniprot accession	*P*-value
12282-5p	−45.7	Novel, function NYD; A	*traW*	Involved in F-pilus assembly. Required for F plasmid conjugative transfer; P18472	0.00029
12207-3p	−45.4	Novel, function NYD; A	*traB*	Conjugal transfer pilus assembly protein; P41067	0.00027
12237-5p	−45.4	Novel, function NYD; A	*aadA4*	Aminoglycoside 3”-adenylyltransferase activity; Q7BPB1	0.00053
12237-5p	−45.2	Novel, function NYD; A	*traW*	Involved in F-pilus assembly. Required for F plasmid conjugative transfer; P18472	0.00033
12267-3p	−44.1	Novel, function NYD; A	*traP*	Conjugal transfer protein V5KCM6	0.00015

*Gene functions based on Uniprot function annotation. Predictive miRNA hybridizations were generated using RNAhybrid version 2.1.2 using the Gallus gallus miRNA database from miRBase database release V22. MFE, minimum free energy; NYD, not yet determined.*

To investigate the role of individual chicken miRNAs in plasmid transfer, mimic miRNA for gga-miR-12267-3p (40-GGGUCGCCCCGGGUCUCGGUGU-61; miRbase accession MIMAT0050029) was synthesized (Creative Biogene). After serial dilution in LB broth, mimic gga-miR-12267-3p was then added at different concentrations (0, 50 ng, or 2 μg per 200 μl reaction) to *E. coli* conjugation assays using donor APEC O2 and recipient HS-4 as described earlier to assess changes in pAPEC-O2-R transfer.

### Statistical Analyses and Binary Heatmap Development

GraphPad Prism software version 6.0 (San Diego, CA) was used to calculate significance between treatment groups via one-way ANOVA followed by Tukey’s test for multiple means comparisons. *P* < 0.05 were considered significant. To summarize binary datasets (i.e., positive or negative) like PCR and antibiotic resistance, R package “d3heatmap” was used to construct binomial heat maps indicating the presence (i.e., green) or absence (i.e., black) of a particular gene, Inc., group, or antibiotic resistance in each *E. coli* isolate.

## Results

### Fecal *E. coli* From P + V Group Exhibited Absence in IncFIB^+^ ColV^+^ Plasmids, Virulence Genes, and Phenotype

**FIGURE 1:**
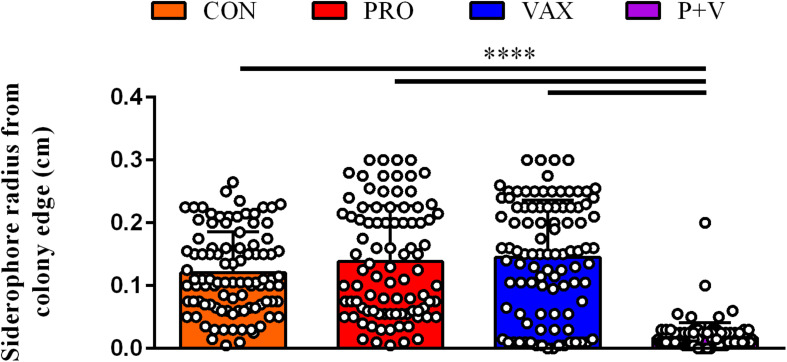
Siderophore production on CAS agar plates by each *E. coli* isolate (dot) per treatment group. Ring radius from individual, bacterial colonies were measured via ruler (cm). ****P < 0.0001.

**FIGURE 2:**
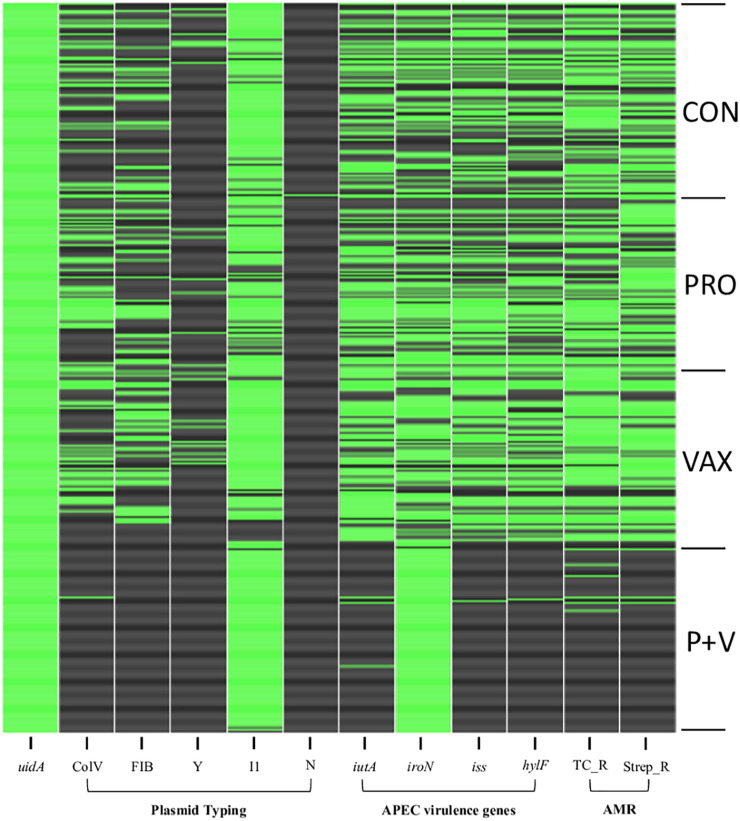
Binary heatmap indicating presence (green) or absence (black) of virulence genes and antibiotic resistance. *uidA*, β-glucuronidase. ColV, *cvaC*-positive. FIB, IncFIB replicon. Y, IncY replicon. I1, IncI1 replicon. N, IncN replicon. *iutA*, aerobactin receptor. *iroN*, salmochelin receptor. *iss*, increased serum survival gene. *hlyF*, hemolysin. TC_R, tetracycline resistance. Strep_R, streptomycin resistance. APEC, avian pathogenic *Escherichia coli.* AMR, antimicrobial resistance.

After plating feces from CON, PRO, VAX, and P + V birds on MacConkey, the vast majority of isolates (≥89% per group) were positive for the *E. coli*-specific marker *uidA* (Bej et al., 1991) (Supplementary Table 1). Proceeding with *E. coli* isolates from each group for screening, we found a significant decrease in siderophore production via CAS agar in P + V *E. coli* isolates (*P* < 0.0001; Figure 1). Similarly, P + V *E. coli* were much more susceptible to tetracycline and streptomycin compared to *E. coli* from other groups (*P* < 0.0001; Figure 2). Using PCR to investigate whether other APEC-associated plasmidic genes were absent in P + V *E. coli*, *iutA, hlyF*, and *iss* were significantly decreased in P + V *E. coli* compared with other groups (*P* < 0.0001; Figure 2). However, the presence of *iroN* was unchanged between groups (Figure 2). Additionally, IncFIB and ColV plasmids were significantly reduced in P + V isolates compared to the other groups (*P* < 0.0001; Figure 2). However, IncI1 plasmids were maintained in all groups, with nearly all P + V isolates tested harboring an IncI1 plasmid. Thus, only fecal *E. coli* from P + V birds exhibited a loss in virulence attributes, associated with an absence of specific plasmid-types (Figure 2).

### Lack of Large Plasmids in Isolates Is Associated With Decreased Ceca smRNA Concentration

**FIGURE 3:**
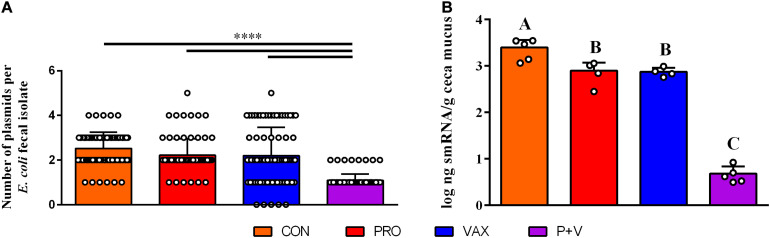
Association of *E. coli* plasmid content **(A)** with smRNA levels in the ceca mucus of the chicken intestine **(B)**. ****P < 0.0001. Differences in letters A, B, and C indicate significant differences between groups (*P* < 0.01).

Using phenol-chloroform plasmid-extraction for each isolate, P + V *E. coli* had significantly less large plasmids (>25 kb) compared to *E. coli* from the other groups (*P* < 0.0001; Figure 3A). Notably, a single ∼100 kb plasmid was generally conserved in all P + V *E. coli* (Supplementary Figure 1).

### Greater Ceca smRNA Concentrations Increased *in vitro* IncF Plasmid Transfer Between *E. coli* Mating Pairs

To identify a mechanism for this loss of plasmids, we hypothesized that host smRNAs may play a role in IncF plasmid transfer. In association with this loss of large plasmids, smRNA concentration in ceca mucus by weight was markedly lower in P + V birds vs. other groups (*P* < 0.01; Figure 3B). These data suggest a potential relationship between *E. coli* plasmid content and host smRNA levels.

**FIGURE 4:**
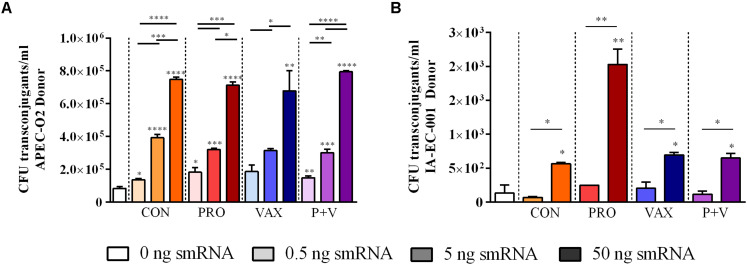
Transconjugant levels of *in vitro E. coli* conjugation assays treated with smRNA, using APEC O2 **(A)** or IA-EC-001 **(B)** as donors. *P < 0.05; **P < 0.01; ***P < 0.001; ****P < 0.0001.

**FIGURE 5:**
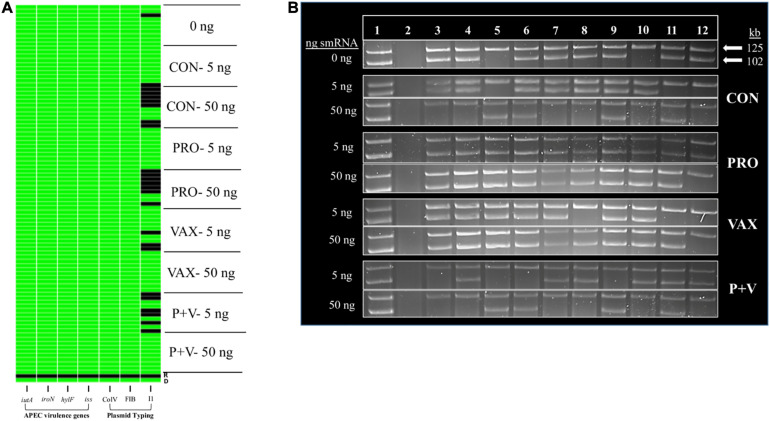
Binary heatmap **(A)** and plasmid profiles **(B)** of *E. coli* transconjugants from IA-EC-001 conjugation assays. *iutA*, aerobactin receptor. *iroN*, salmochelin receptor. *hlyF*, hemolysin. *iss*, increased serum survival. ColV, FIB, and I1 indicate plasmid type. D **(A)** or 1 **(B)**, IA-EC-001 donor. R **(A)** or 2 **(B)**, HS-4 recipient. 3–12 **(B)**, *in vitro* transconjugants.

Using *in vitro* conjugation assays with and without smRNA pooled within each group, we found that smRNA concentrations were positively associated with HS-4 transconjugant yields (*P* < 0.01) using APEC O2 (Figure 4A), carrying TC_*R*_ carried on the IncF plasmid pAPEC-O2-R (Johnson et al., 2006) or VAX isolate IA-EC-001 (IncFIB^+^ ColV^+^, this study; Figure 4B) as donor strains. Furthermore, this effect was generally independent of the treatment group, as this pattern was consistent in conjugation assays with smRNA from all treatment groups (Figure 4). Importantly, growth of donor or recipient strains was independent of smRNA concentration (Supplementary Figure 2), suggesting that these smRNAs had a specific effect on conjugation and not *E. coli* growth during this time frame. To characterize the IA-EC-001 plasmids being transferred in this assay, transconjugants from each smRNA dilution per group (*n* = 10) were isolated and genotyped via PCR. Transfer of TC_*R*_ from the IA-EC-001 isolate to HS-4 recipients was consistent with transfer of the genes *iutA*, *iroN, hlyF*, and *iss* (Figure 5A). Furthermore, this was linked with IncF1B and ColV-plasmid typing (Figure 5A), suggesting TC_*R*_ and these genes were being carried on a single IncFIB^+^ ColV^+^ plasmid. This assumption was further supported by consistent identification of a ∼125 kb plasmid found in every single transconjugant (Figure 5B). Finally, though a high proportion of transconjugants also received a ∼102 kb IncI1 plasmid (Figure 5), this was not consistently found among transconjugants as were the previous biomarkers.

### Host miRNA Species Predicted to Target pAPEC-O2-R Genes

To determine whether specific smRNAs may affect IncF plasmid transfer, we first used RNAHybrid to predict whether chicken miRNAs could hybridize with pAPEC-O2-R genes. RNAHybrid analysis resulted in 74 bindings within known coding sequences with *P*-values below 0.001 when hybridized with the large antimicrobial resistance plasmid pAPEC-O2-R (Supplementary Table 3). The top five high affinity bindings demonstrated minimum free energies of less than -44 kcal/mol and *P*-values below 0.0006 (Table 2). Although functions for these miRNA have yet to be determined (Warnefors et al., 2017), their predicted gene targets are largely involved in F-pilus assembly and conjugal transfer proteins (Table 2). Using RT-qPCR to identify these miRNAs in our samples, we found that gga-miR-12267-3p was the only detectable miRNA species in all groups (Supplementary Figure 3A). However, adding synthetic gga-miR-12267-3p miRNA to pAPEC-O2-R conjugation assays did not alter donor (APEC O2; Supplementary Figure 3B), recipient (HS-4; Supplementary Figure 3C), nor transconjugant abundances *in vitro* (Supplementary Figure 3D).

## Discussion

We report that live bacterial prophylactics, specifically the combination of probiotics and live *Salmonella* vaccine used in this study, reduced virulence trait and plasmid-containing *E. coli* in the chicken gut. The vast number of fecal *E. coli* isolates in this study possessed APEC virulence factors, suggesting there is a competitive-advantage for possessing these genes in the intestine. However, there was a marked loss of iron-acquisition ability in P + V *E. coli* compared to the other groups. This loss was highly associated with the loss of *iutA*, which encodes the receptor for the aerobactin system. Notably, this system is a highly effective mode of iron acquisition compared to other systems and is crucial for inter-bacterial competition in the intestine (Garcia et al., 2011; Deriu et al., 2013). ExPEC, which cause extraintestinal diseases like urinary tract infections in mammalian models and colibacillosis in avian species (Mellata, 2013), are similar to commensal *E. coli* in genetic composition (Grozdanov et al., 2004) and their avid colonization of the animal intestinal tract (Ewers et al., 2009; Diard et al., 2010; Meador et al., 2014; Stromberg et al., 2017).

The absence of these virulence genes and phenotypes like AMR in P + V *E. coli* is directly related to the absence of IncFIB and ColV plasmids. These plasmid types carry virulence factors like iron acquisition systems (i.e., aerobactin and salmochelin), tetracycline and streptomycin-resistance, serum resistance (i.e., *iss*), and hemolysins (i.e., *hylF*) for APEC (Johnson et al., 2005; Johnson and Nolan, 2009; Mellata et al., 2009, 2010). ColV plasmids classified by their possession of the *cvaC* gene, which encodes the antimicrobial compound colicin V and targets other *Enterobacteriaceae* (Cascales et al., 2007). Given that APEC depend on plasmid virulence factors like iron acquisition and serum resistance for infection (Mellata et al., 2010) upon fecal aerosolization in young birds (Duan et al., 2008; Chien et al., 2011), the reduction of these virulence attributes in commensal *E. coli* would dramatically lower risks of contracting colibacillosis. Interestingly, this prophylactic combination also enhanced systemic clearance of APEC infection *in vivo* via increased bactericidal responses in blood (Redweik et al., 2020), suggesting P + V birds are protected from APEC infection via multiple mechanisms.

P + V treatment uniquely reduced smRNA in the ceca mucus, and intestinal smRNA concentrations were positively associated with IncF plasmid transfer *in vitro*. This is the first study to identify smRNA as a potential mediator for plasmid transfer in the animal intestine. Characterizing microbe-microbe interactions in the gut and how host genetics and factors drive these interactions has been a major gap in microbiome research (NIH Human Microbiome Portfolio Analysis Team, 2019). Host signals like catecholamines increase plasmid transfer *in vitro*, theoretically to promote bacteria to exchange genes during periods of acute stress in their host (Peterson et al., 2011). In mice, smRNA species like miRNA are released via vesicles from epithelial cells into the gut lumen under homeostatic conditions (Liu et al., 2016). Thus, we hypothesized that host miRNA may be a signal intestinal bacteria use to mediate plasmid transfer. Although several *Gallus gallus* miRNAs were predicted to interact with pAPEC-O2-R genes essential for the function and fertility of plasmid replication and conjugative transfer (Maneewannakul et al., 1992), we only detected one of these species, gga-miR-12267-3p, in our samples. Although gga-miR-12267-3p was predicted to bind to *traP*, a conjugal transfer protein carried on pAPEC-O2-R, its individual role in promoting plasmid transfer was not demonstrated in our conditions tested, suggesting the role of smRNAs may be multifactorial. Still, the question remains whether the smRNAs responsible for regulating plasmid transfer are of host or bacterial origin. For example, *E. coli* use *finP*, an antisense, non-coding, 79-nucleotide RNA molecule, which forms a complex with FinO to suppress plasmid transfer intracellularly (Koraimann et al., 1996; Torreblanca et al., 1999). Future studies will determine whether individual smRNA species (of host and/or bacterial origin) or a synergism between smRNAs are required to promote IncF plasmid transfer. Importantly, this study did not fully elucidate the role of a specific miRNA in intestinal IncF plasmid transfer *in vivo*. Future studies will use *in vivo* conjugation transfer experiments to confirm that smRNAs drive IncF plasmid transfer in the chicken intestine.

Uniquely, a single IncI1 plasmid and salmochelin receptor *iroN* remained highly conserved in P + V fecal *E. coli*, and IncI1 *in vitro* plasmid transfer by isolate IA-EC-001 was largely unaffected by smRNA concentration. The salmochelin operon is commonly found on IncI1 plasmids (Kaldhone et al., 2019), which suggests this conserved IncI1 plasmid may be responsible for this observed-*iroN* maintenance in P + V isolates. This suggests that intestinal smRNAs specifically target genes on IncF plasmids and do not affect the transfer of IncI1 plasmids. Reasons underlying the conservation of this particular IncI1 plasmid in P + V isolates are unclear. However, since IncF and IncI1 plasmids have unique mechanisms for replication and transfer between bacterial hosts (del Solar et al., 1998; Zatyka and Thomas, 1998), intestinal smRNAs could specifically target IncF genes involved in these processes, although this still remains to be confirmed.

In conclusion, our findings suggest the combination of these live prophylactics, despite not being specifically designed to target AMR and virulence plasmids, reduced abundances of IncF virulence plasmids and associated ExPEC characteristics in fecal *E. coli*, by potentially reducing intestinal smRNA levels. This suggests that combining these probiotics and live vaccines may reduce antimicrobial resistance by reducing IncF plasmid transfer between intestinal *E. coli* as well as directly antagonizing *Enterobacteriaceae* colonization and infection (Redweik et al., 2019, 2020). However, it should be noted that these samples were taken within a specific time window pre-lay and that these pullets were from the same flock. It is possible that changes in smRNA levels over time may occur (Noren Hooten et al., 2013) and thus could likely result in changes to bacterial conjugation in the chicken intestine. Furthermore, the environment plays a major role in microbiome development in poultry animals (Kers et al., 2018). Thus, this mechanism may be permissible to pullets exposed to one particular microbiome but perhaps not another. Current work is underway to understand these nuances as well as identify smRNA molecular mechanisms which drive plasmid transfer.

## Data Availability Statement

The original contributions presented in the study are included in the article/Supplementary Material, further inquiries can be directed to the corresponding author/s.

## Ethics Statement

The animal study was reviewed and approved by the Iowa State University Institutional Animal Care and Use Committee, log #1-16-8159-G.

## Author Contributions

GR and MM conceived, designed the experiments, and wrote the manuscript. GR, RH, MH, and LO performed the experiments and analyzed the data. GR, RH, MH, LO, and MM revised the manuscript. MM contributed to reagents, materials, and analysis tools. All authors read and approved the final manuscript.

## Conflict of Interest

The authors declare that the research was conducted in the absence of any commercial or financial relationships that could be construed as a potential conflict of interest.

## Footnotes

1 ftp://mirbase.org/pub/mirbase/CURRENT/mature.fa.gz

2 https://bibiserv.cebitec.uni-bielefeld.de/rnahybrid

## Supplementary Material

Supplementary Figure 1 Plasmid profiles of strains in 0.5% agarose TAE gel. Gel A: 1, *E. coli* ladder 39R681; 2, *E. coli* HS-4 (negative control); 3–12, individual P + V isolates. Gel B: 1, *E. coli* ladder 39R681; 2–12, individual P + V isolates. Gel C: 1, *E. coli* ladder 39R681; 2–12, individual P + V isolates.

Supplementary Figure 2 Donor **(A,C)** and recipient **(B,D)** levels of *in vitro E. coli* conjugation assays treated with smRNA.

Supplementary Figure 3 **(A)** qPCR reads for chicken miRNAs predicted to hybridize with pAPEC-O2-R target genes (see Table 2). **(B–D)** Levels of donor (APEC O2, **B**), recipient (HS-4, **C**) and transconjugants **(D)** from *in vitro E. coli* conjugation assays treated with synthetic mimic gga-miR-12267-3p miRNA at different concentrations (0, 50 ng, and 2 μg per 200 μl reactions). ^∗^*P* < 0.05.

Supplementary Table 1 Summary of PCR primers used in this study for virulence gene and plasmid replicon detection.

Supplementary Table 2 Summary of *uidA* detection in fecal isolates from 100 randomly selected colonies from each treatment group. 1 indicates positive, 0 indicates negative.

Supplementary Table 3 Complete RNAHybrid predictive bindings output, gene targets, and gene names for all significant interactions (*P* < 0.001).
