# Supplementary figures and images for: Evaluation of Live Bacterial Prophylactics to Decrease IncF Plasmid Transfer and Association With Intestinal Small RNAs
Source: Front Microbiol. 2021 Jan 14;11:625286. doi: 10.3389/fmicb.2020.625286 (PMC7840957; doi:10.3389/fmicb.2020.625286)

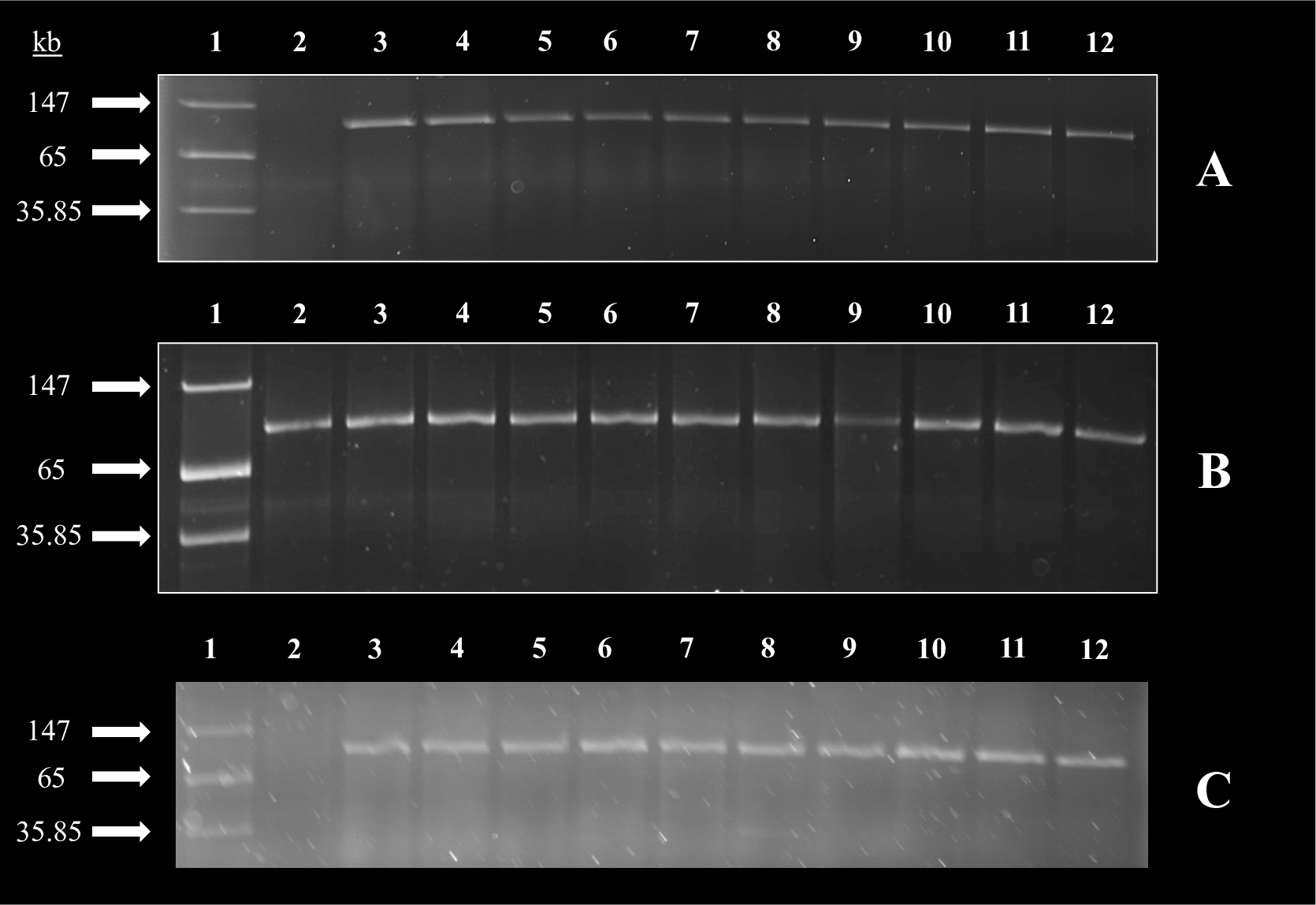

Supplement: Supplementary Figure 1 — Plasmid profiles of strains in 0.5% agarose TAE gel. Gel A: 1, E. coli ladder 39R681; 2, E. coli HS-4 (negative control); 3–12, individual P + V isolates. Gel B: 1, E. coli ladder 39R681; 2–12, individual P + V isolates. Gel C: 1, E. coli ladder 39R681; 2–12, individual P + V isolates. [file Image_1.tif]

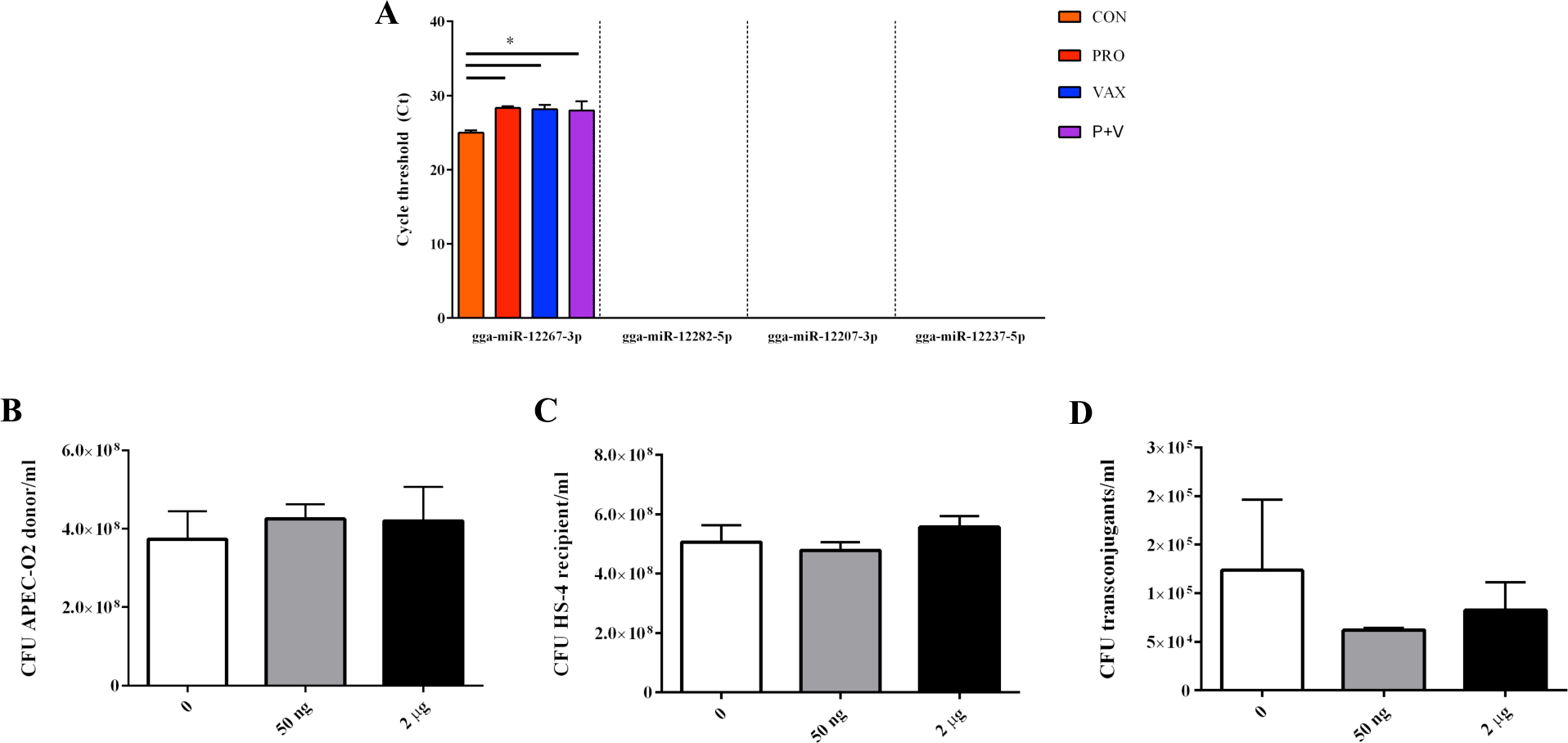

Supplement: Supplementary Figure 3 — (A) qPCR reads for chicken miRNAs predicted to hybridize with pAPEC-O2-R target genes (see Table 2). (B–D) Levels of donor (APEC O2, B), recipient (HS-4, C) and transconjugants (D) from in vitro E. coli conjugation assays treated with synthetic mimic gga-miR-12267-3p miRNA at different concentrations (0, 50 ng, and 2 μg per 200 μl reactions). ∗P < 0.05. [file Image_3.tif]
