# Supplementary material for: Evaluation of Live Bacterial Prophylactics to Decrease IncF Plasmid Transfer and Association With Intestinal Small RNAs
Source: Front Microbiol. 2021 Jan 14;11:625286. doi: 10.3389/fmicb.2020.625286 (PMC7840957; doi:10.3389/fmicb.2020.625286)
Supplement: Supplementary Table 1 — Summary of PCR primers used in this study for virulence gene and plasmid replicon detection. [file Table_1.docx]

| **Gene Target** | **Forward Primer (5’-3’)** | **Reverse Primer (5’-3’)** | **Reference** |
| --- | --- | --- | --- |
| *uidA* | TGGTAATTACCGACGAAAACGGC | ACGCGTGGTTACAGTCTTGCG | OO |
| *iss* | CAGCAACCCGAACCACTTGATG | AGCATTGCCAGAGCGGCAGAA | PP |
| *iutA* | GGCTGGACATCATGGGAACTGG | CGTCGGGAACGGGTAGAATCG | PP |
| *iroN* | AATCCGGCAAAGAGACGAACCGCCT | GTTCGGGCAACCCCTGCTTTGACTTT | PP |
| *hylfA* | GGCCACAGTCGTTTAGGGTGCTTACC | GGCGGTTTAGGCATTCCGATACTCAG | PP |
| *cvaC* | CACACACAAACGGGAGCTGTT | CTTCCCGCAGCATAGTTCCAT | QQ |
| **Replicon** |  |  |  |
| Panel 1: |  |  |  |
| B/O | GCGGTCCGGAAAGCCAGAAAAC | TCTGCGTTCCGCCAAGTTCGA | E |
| FIC | GTGAACTGGCAGATGAGGAAGG | TTCTCCTCGTCGCCAAACTAGAT | E |
| A/C | GAGAACCAAAGACAAAGACCTGGA | ACGACAAACCTGAATTGCCTCCTT | E |
| P | CTATGGCCCTGCAAACGCGCCAGAAA | TCACGCGCCAGGGCGCAGCC | E |
| T | TTGGCCTGTTTGTGCCTAAACCAT | CGTTGATTACACTTAGCTTTGGAC | E |
| Panel 2: |  |  |  |
| K/B | GCGGTCCGGAAAGCCAGAAAAC | TCTTTCACGAGCCCGCCAAA | E |
| W | CCTAAGAACAACAAAGCCCCCG | GGTGCGCGGCATAGAACCGT | E |
| FIIA | CTGTCGTAAGCTGATGGC | CTCTGCCACAAACTTCAGC | E |
| FIA | CCATGCTGGTTCTAGAGAAGGTG | GTATATCCTTACTGGCTTCCGCAG | E |
| FIB | GGAGTTCTGACACACGATTTTCTG | CTCCCGTCGCTTCAGGGCATT | E |
| Y | AATTCAAACAACACTGTGCAGCCTG | GCGAGAATGGACGATTACAAAACTTT | E |
| Panel 3: |  |  |  |
| I1 | CGAAAGCCGGACGGCAGAA | TCGTCGTTCCGCCAAGTTCGT | E |
| Frep | TGATCGTTTAAGGAATTTTG | GAAGATCAGTCACACCATCC | E |
| X | AACCTTAGAGGCTATTTAAGTTGCTGAT | TGAGAGTCAATTTTTATCTCATGTTTTAGC | E |
| HI1 | GGAGCGATGGATTACTTCAGTAC | TGCCGTTTCACCTCGTGAGTA | E |
| N | GTCTAACGAGCTTACCGAAG | GTTTCAACTCTGCCAAGTTC | E |
| HI2 | TTTCTCCTGAGTCACCTGTTAACAC | GGCTCACTACCGTTGTCATCCT | E |
| L/M | GGATGAAAACTATCAGCATCTGAAG | CTGCAGGGGCGATTCTTTAGG | E |
