# Supplementary material for: Evaluation of Live Bacterial Prophylactics to Decrease IncF Plasmid Transfer and Association With Intestinal Small RNAs
Source: Front Microbiol. 2021 Jan 14;11:625286. doi: 10.3389/fmicb.2020.625286 (PMC7840957; doi:10.3389/fmicb.2020.625286)
Supplement: Supplementary Table 2 — Summary of uidA detection in fecal isolates from 100 randomly selected colonies from each treatment group. 1 indicates positive, 0 indicates negative. [file Table_2.docx]

| **Treatment**  **Group** | **% *uidA*-positive** |
| --- | --- |
| CON | 97 |
| PRO | 89 |
| VAX | 93 |
| P+V | 100 |
